# Clinical characteristics of congenital lamellar cataract and myopia in a Chinese family

**DOI:** 10.1042/BSR20191349

**Published:** 2020-02-14

**Authors:** Qing Liu, Siquan Zhu

**Affiliations:** Department of Ophthalmology, Beijing AnZhen Hospital, Capital Medical University, Beijing 100029, China

**Keywords:** Chinese family, congenital lamellar cataract, myopia

## Abstract

To investigate the clinical characteristics and the genetic defect in a Chinese family with congenital lamellar cataract with myopia. Three generations of a single family were recruited in the present study. A detailed family history and clinical data were recorded. A total of 100 unrelated ethnically matched controls without family history of congenital cataracts and myopia were also recruited. Genomic DNA was extracted from peripheral blood leukocytes. The sequencing of candidate genes was performed to screen out the disease-causing mutation. The effects of amino acid changes on the structure of proteins were predicted by bioinformatics analysis. Affected individuals presented lamellar lens opacities and myopia. Direct sequencing revealed a heterozygous c. 34 C>T variation in the αA-crystallin protein (*CRYAA*) gene, which resulted in the replacement of a highly conserved arginine by cystine at codon 12 (p.R12C). This mutation co-segregated with all affected individuals and was not observed in unaffected members or the 100 normal controls. Bioinformatic analysis showed that a highly conserved region was located around Arg12, an increase in local hydrophobicity was shown around the substitution site and the secondary structure of the mutant *CRYAA* protein has been changed. This is the case of a congenital lamellar cataract phenotype with myopia associated with the mutation of Arg12Cys (p.R12C) in *CRYAA*. Our finding confirms the high rate of mutations at this dinucleotide. In addition, these results demonstrate a myopia susceptibility locus in this region, which might also be associated with the mutation in *CRYAA*.

## Introduction

Congenital cataract is a clinically and genetically heterogeneous lens disease characterized by significant visual impairment and blindness in childhood [1,2]. The incidence is 0.6/10,000 to 6/10,000 [3]. Cataracts can be isolated or occur in association with metabolic diseases or genetic syndromes. About one-third cataracts are genetic [3]. A majority of congenital cataracts are single gene disorders. Autosomal dominant inheritance is the most common mode of congenital cataract [3]. Autosomal recessive and X-linked fashion have also been reported [4]. According to morphology, cataracts can be classified into different categories, including nuclear, lamellar, cortical, polar, sutural, pulverulent, cerulean, coralliform, and whole lens, as well as other minor subtypes [3].

Up to date, more than 34 loci and 18 genes on different chromosomes have been identified to be associated with autosomal dominant congenital cataract (ADCC) [5,6]; moreover, about half of them have mutations in crystallins, a quarter have mutations in connexins, and the remainder is evenly divided into intrinsic membrane proteins, intermediate filament proteins, transcription factors and other genes [7]. Currently, genes with mutations associated with mixed lamellar cataracts include four groups. The first group is crystalline genes (*CRYAA, CRYAB, CRYBA1, CRYBB2, CRYGC, CRYGD*). The second group is encoding membrane transport proteins, including *GJA3* and *GJA8.* The third group is beaded filament structural protein 2 (*BFSP2*), encoding cytoskeletal protein. The last group is heat shock transcription factor 4 (*HSF4*), encoding transcription factors [3,7]. Therefore, it is rational to consider these genes as the top list of candidate genes for screening studies in congenital lamellar cataracts.

In the present study, we applied a function candidate testing approach to the known lamellar cataract-causing genes in a Chinese family. A R→C mutation in *CRYAA* that co-segregated with the disease phenotype was identified to be responsible for ADCC, which provides a possible mechanism of action for the mutant gene. This mutation has previously been described in Danish and Hong Kong families, which were both shown microcornea-cataract [8,9]. However, isolated lamellar l cataract such as those reported here has not been identified in the previous reports.

## Materials and methods

### Clinical evaluation and DNA specimens

A three-generation family with autosomal dominant lamellar cataract and myopia from Henan province, China, were recruited at Beijing Tongren Hospital, the Capital Medical University. Both affected and unaffected individuals of the family underwent detailed ophthalmic examinations including visual acuity, slit lamp examination, ultrasonography, fundus examination, and intraocular pressure measurement. The phenotypes were documented by slit lamp photography. A total of 100 unrelated ethnically matched controls with no family history of congenital cataracts and myopia were also recruited. They were given complete ophthalmologic examinations as the study subjects of the cataract family and confirmed without eye diseases except for senile cataracts. Individuals with one of the following three criteria were considered to be affected with myopia: (1) cycloplegic refraction of −1.00 D spherical equivalent or lower in individuals <30 years old; (2) manifest refraction of −1.00 D spherical equivalent or lower in individuals ≥30 years old, or (3) axial length >26 mm (an extension of 1 mm would generally result in myopia of −3.00 D, the normal range of axial length in Chinese is 23.5–24.5 mm). With the consent of all participants, the peripheral venous blood of all participants was collected and genomic DNA was extracted using a QIAamp DNA kit (Qiagen, Valencia, CA) in accordance with the manufacturer’s instructions. All patients had provided informed consents before participating in the present study. The present study was conducted in accordance with the tenets of the Declaration of Helsinki and approved by the ethics committees for medical research at the Capital Medical University in Beijing, China.

### Mutation screening

Eleven candidate genes, including *CRYBA1* (GenBank NM_005208), *CRYBB2* (GenBank NM_000496), *MIP* (GenBank NM_012064.3), and *BFSP2* (GenBank NM_003571), are highly expressed in the lens and have been considered as candidate genes for hereditary lamellar cataracts [3,7]. Mutation screening was performed in these candidate genes. We amplified each exon and intron–exon junction of the genes with previously published primer sequences (Table 1) [10] by polymerase chain reaction (PCR). Each reaction mix (25 μl) contained 20 ng of genomic DNA, 1× PCR buffer, 1.5 mM MgCl_2_, 0.2 mM dNTPs, 0.5 μM each of forward and reverse primers and 2.5U of Taq DNA polymerase (Qiagen). A PCR program was performed for DNA amplifying: 95°C for 3 min; followed by 35 cycles at 95°C for 30 s, 57–63°C for 30 s (annealing temperature depending on different primer); 72°C for 45 s; and a final extension at 72°C for 7 min. The PCR products of the proband and one unaffected member were sequenced using an ABI3730 Automated Sequencer (PE Biosystems, Foster City, CA). The sequencing results were analyzed using Chromas 2.33 and compared with the reference sequence in the NCBI database. Then, we screened the mutation in *CRYAA* from the sample of the family members and 100 ethnically matched controls to confirm the mutation.

**Table 1:** Primers for PCR

Name	Forward (5′-3′)	Reverse (3′-5′)
CRYAA-1	AGCAGCCTTCTTCATGAGC	CAAGACCAGAGTCCATCG
CRYAA-2	GGCAGGTGACCGAAGCATC	GAAGGCATGGTGCAGGTG
CRYAA-3	GCAGCTTCTCTGGCATGG	GGGAAGCAAAGGAAGACAGA
CRYAB-1	AACCCCTGACATCACCATTC	AAGGACTCTCCCGTCCTAGC
CRYAB-2	CCATCCCATTCCCTTACCTT	GCCTCCAAAGCTGATAGCAC
CRYAB-3	TCTCTCTGCCTCTTTCCTCA	CCTTGGAGCCCTCTAAATCA
CRYBA1-1	GGCAGAGGGAGAGCAGAGTG	CACTAGGCAGGAGAACTGGG
CRYBA1-2	AGTGAGCAGCAGAGCCAGAA	GGTCAGTCACTGCCTTATGG
CRYBA1-3	AAGCACAGAGTCAGACTGAA	CCCCTGTCTGAAGGGACCTG
CRYBA1-4	GTACAGCTCTACTGGGATTG	ACTGATGATAAATAGCATGAA
CRYBA1-5	GAATGATAGCCATAGCACTAG	TACCGATACGTATGAAATCTG
CRYBA1-6	CATCTCATACCATTGTGTTGAG	GCAAGGTCTCATGCTTGAGG
CRYBB2-1	GTTTGGGGCCAGAGGGGAGT	TGGGCTGGGGAGGGACTTTC
CRYBB2-2	CCTTCAGCATCCTTTGGGTTC	GCAGTTCTAAAAGCTTCATCA
CRYBB2-3	GTAGCCAGGATTCTGCCATAG	GTGCCCTCTGGAGCATTTCAT
CRYBB2-4	GGCCCCCTCACCCATACTCA	CTTCCCTCCTGCCTCAACCTA
CRYBB2-5	CTTACCCTTGGGAAGTGGCAA	TCAAAGACCCACAGCAGACA
CRYGC-1	TGCATAAAATCCCCTTACCG	CCTCCCTGTAACCCACATTG
CRYGC-2	TGGTTGGACAAATTCTGGAAG	CCCACCCCATTCACTTCTTA
CRYGD-1	CAGCAGCCCTCCTGCTAT	GGGTCCTGACTTGAGGATGT
CRYGD-2	GCTTTTCTTCTCTTTTTATTTC	AAGAAAGACACAAGCAAATC
GJA3-1	CGGTGTTCATGAGCATTTTC	CTCTTCAGCTGCTCCTCCTC
GJA3-2	GAGGAGGAGCAGCTGAAGAG	AGCGGTGTGCGCATAGTAG
GJA3-3	TCGGGTTCCCACCCTACTAT	TATCTGCTGGTGGGAAGTGC
GJA8-1	CCGCGTTAGCAAAAACAGAT	CCTCCATGCGGACGTAGT
GJA8-2	GCAGATCATCTTCGTCTCCA	GGCCACAGACAACATGAACA
GJA8-3	CCACGGAGAAAACCATCTTC	GAGCGTAGGAAGGCAGTGTC
GJA8-4	TCGAGGAGAAGATCAGCACA	GGCTGCTGGCTTTGCTTAG
BFSP2(1a)	AATGCACAAACCCAAATGGT	AGGCCCTGSSGACACT
BFSP2(1a)	GAGAGGCGAGTGGTAGTGGA	GGCCTCAGCCTACTCACAAC
BFSP2(2)	TGCAGACAGAGCATTTCCAC	GAGGGGTGTGAGCTGGATAA
BFSP2(3)	GCTGCAATTGCCTTCATTTT	GGGTAACCTGACCCAACTTCA
BFSP2(4)	TCTGTGAAGCCTGTGTCTGG	CCCGGCCTCAATTATTCTTT
BFSP2(5)	ACCCAGGAGGAGGAGGTTGT	GGGAATCCCCTGGAAACTAA
BFSP2(6)	GGGGAATAGTCCAGGCTACC	ATGGGTGCCTATGTGAGAGGG
BFSP2(7)	TTGTTCCAAAGGCCAGATTC	CACTCAAGGGAATCCTTCCA
HSF4-1	CATCCCATCCAGCCAGCCTTT	GGGCATGGGTGTTCACTGACG
HSF4-2	CCTCGACCCATATCCCCGTAA	GCAGGAGCAAGGCAGGCAGT
HSF4-3	GCGGGAATGAGCAAAGAGGA	GCCAAGGCAGGAGAGAGGAA
HSF4-4	TCCCCAGCCTCGCCATTCT	CCCGGTGAAGGAGTTTCCAG
HSF4-5	GCTGGGGCCTGAGGGAG	GGCTTCCATCTTCTCTTCCTTT

### Bioinformatics analysis

The CLC Free Workbench 5.0 software (CLC bio, Aarhus, Denmark) was used to align the protein sequences from several different species. The comparison of hydrophobicity between wild-type and mutant type was analyzed by ProtScale. Garnier–Osguthorpe–Robson (GOR) software was used to predict the effect of the mutation on the secondary structure of *CRYAA*.

## Results

### Clinical characteristics

**Figure 1:**
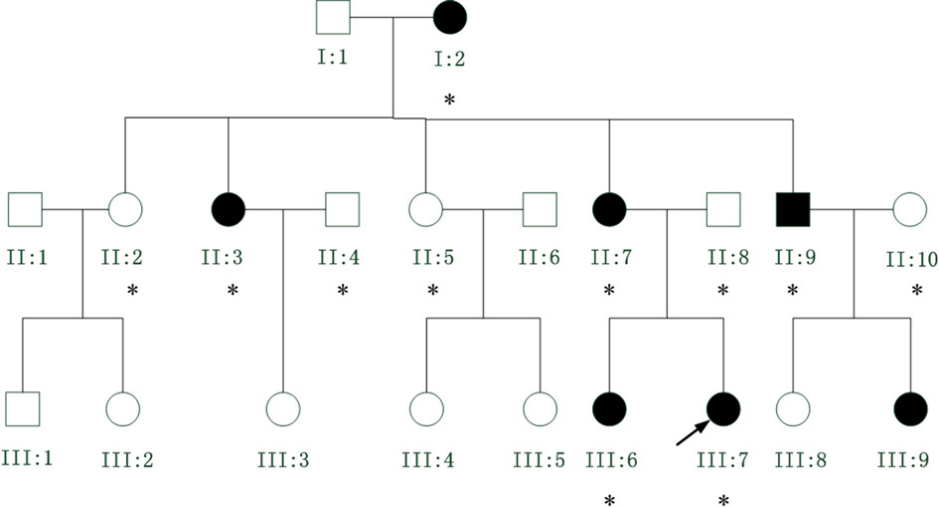
A three-generation Chinese family with clear diagnosis of ADCC and myopia

**Figure 2:**
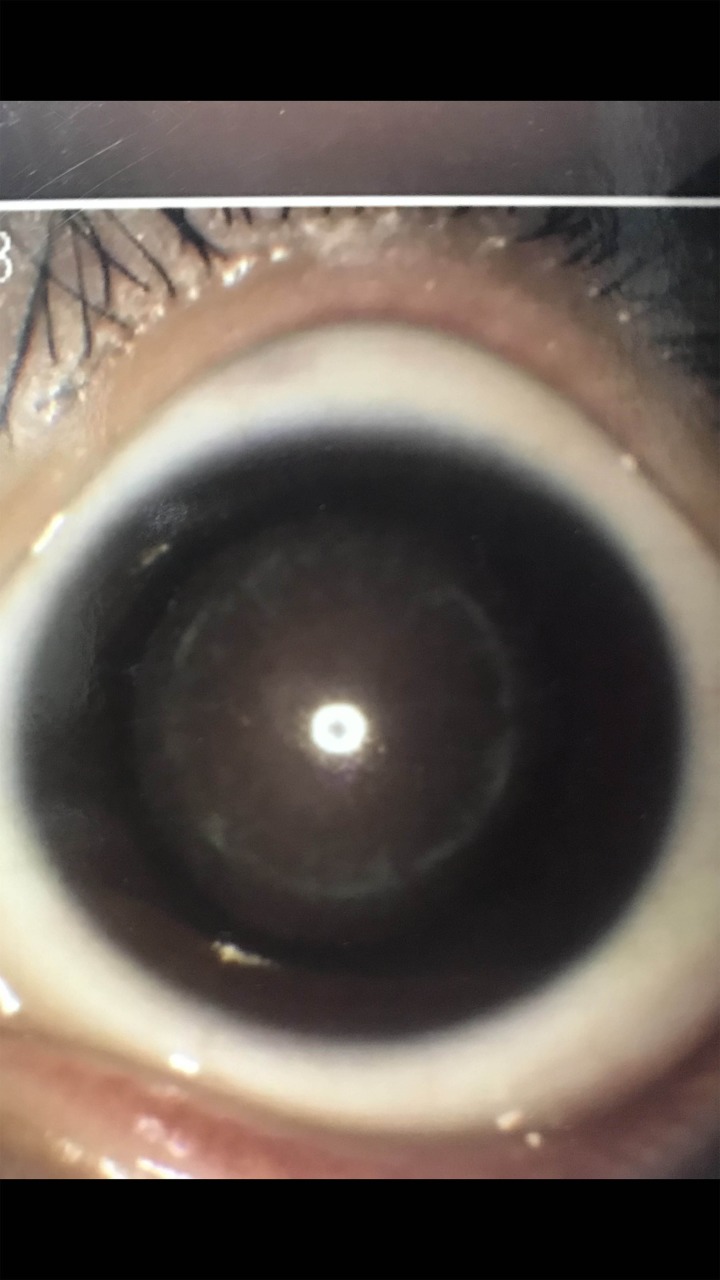
The patient with amellar opacity cataracts

A three-generation Chinese family with clear diagnosis of ADCC and myopia was identified (Figure 1). All affected individuals in this family had lamellar opacity cataracts (Figure 2) in total 11 family members (six affected and five unaffected) participated in the study (Table 2). The proband was a 16-year-old girl (III:7), whose vision had decreased since birth. She was diagnosed with bilateral cataract at the age of four and myopia at 7 years old. An affected member (III:6), the 22-year-old sister of the proband, was diagnosed with bilateral cataract and myopia at the age of seven, and underwent right eye’s cataract extraction at the age of ten. Another affected member (II:9), whose phenotype was similar with the proband, had obvious lamellar and cortical opacity. According to the medical records, other affected individuals diagnosed with bilateral lamellar cataract and myopia have been treated with cataract extraction in different years.

**Table 2:** Clinical data of affected members in this family

ID:	Gender	Age at first symptom	Visual acuity		Phenotype	Axial length	
			OD	OS		OD	OS
I:2	Female	NA	0.08	0.08	NA	NA	NA
II:2	Female	At birth	0.2	0.3	Lamellar	NA	NA
II:7	Female	At birth	0.3	0.3	Lamellar	27.32	26.82
II:9	Male	At birth	0.1	0.2	Lamellar, cortical	27.21	27.10
III:6	Female	At birth	0.4	0.3	Lamellar	26.16	25.53
III:7	Female	At birth	0.3	0.4	Lamellar	25.23	25.21
III:9	Female	At birth	0.4	0.5	Lamellar	NA	NA

The visual acuity column(s) indicates the current visual acuity of the affected members, “NA” indicates not available.

Myopia was present in all affected members, the ocular axial length was extended in the four affected individuals for whom records are available (Table 2). Thus, the myopia is more likely to be of an axial nature rather than being secondary to lens. Most patients experience decreased visual acuity at birth, although it is unclear whether it is caused by lamellar cataract or the accompanying myopia. There was no family history of other ocular or systemic diseases.

### Mutation analysis

**Figure 3:**
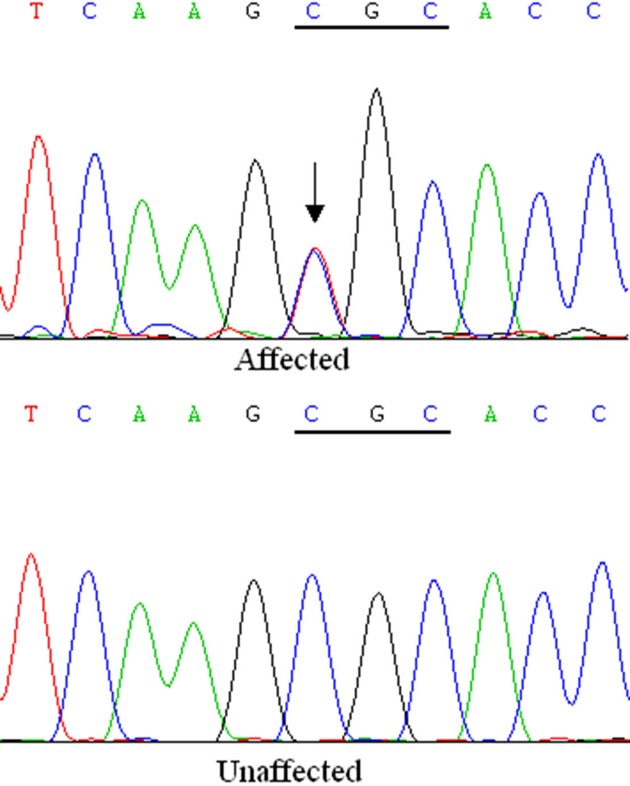
A heterozygous c. 34C >T variation of *CRYAA* in the affected individuals

Through direct sequencing of the coding regions of the candidate genes, we identified a heterozygous c. 34C >T variation of *CRYAA* in the affected individuals (Figure 3). It resulted in a substitution of arginine to cystine at codon 12 (p. R12C). The substitution was not found in any of the unaffected family members or in the 100 participants in the control group. We did not find any other mutations in the family except for a few nonpathogenic single nucleotide polymorphisms (SNPs).

### Bioinformatics analysis

**Figure 4:**
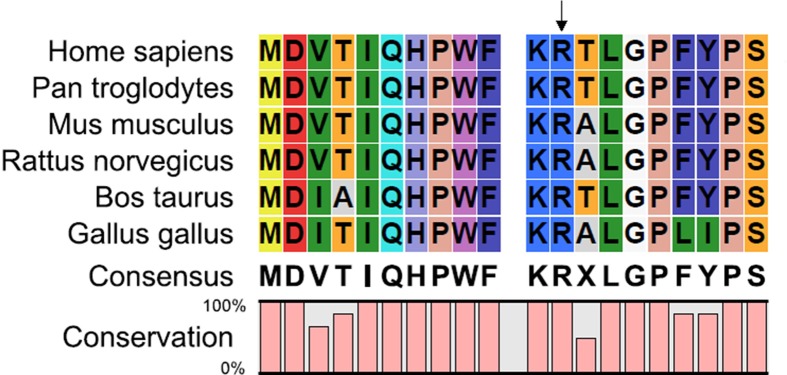
The place where the mutation occurred was located within a phylogenetically conserved region by multiple-sequence alignment Arg12 was a highly conserved residue.

**Figure 5:**
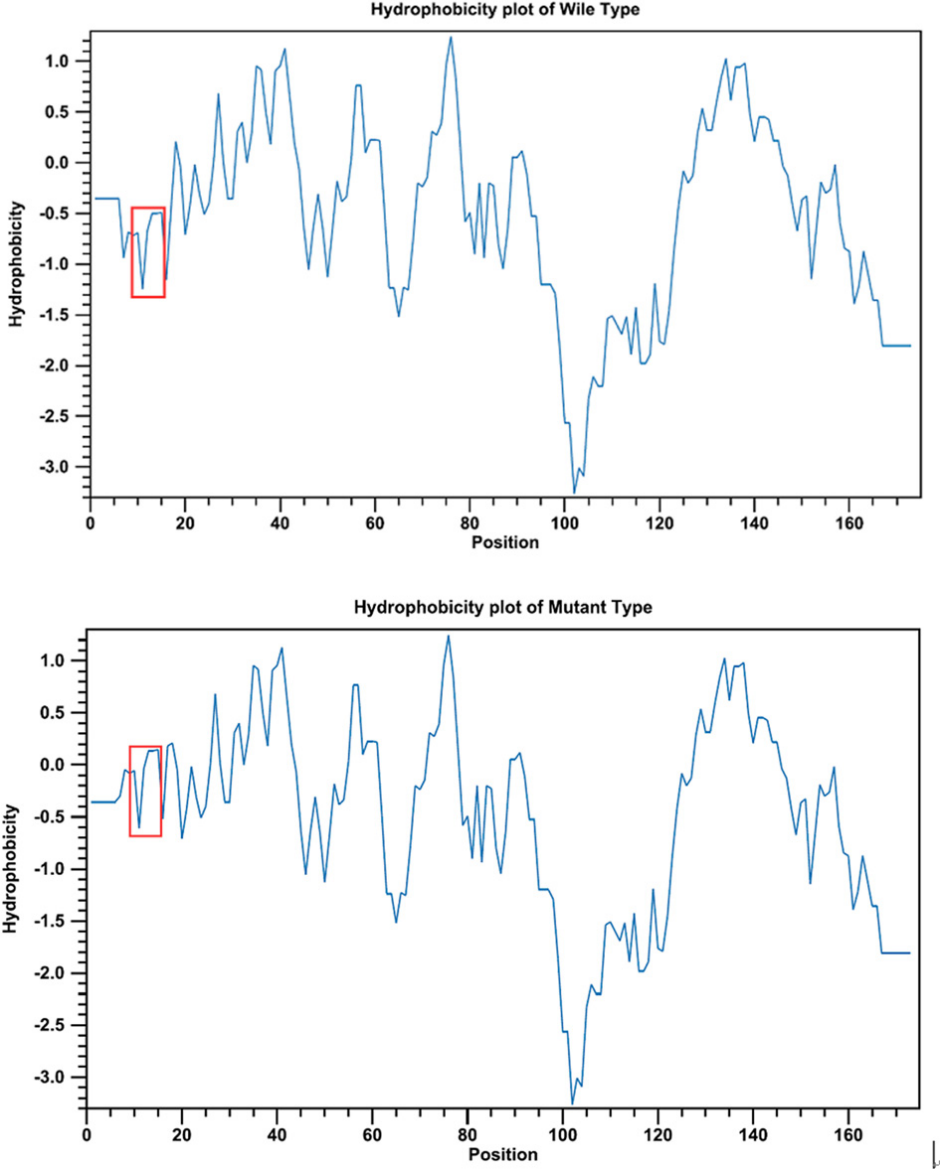
Substitution in the *CRYAA* protein increases the hydrophobicity between amino acids 8 and 16

**Figure 6:**
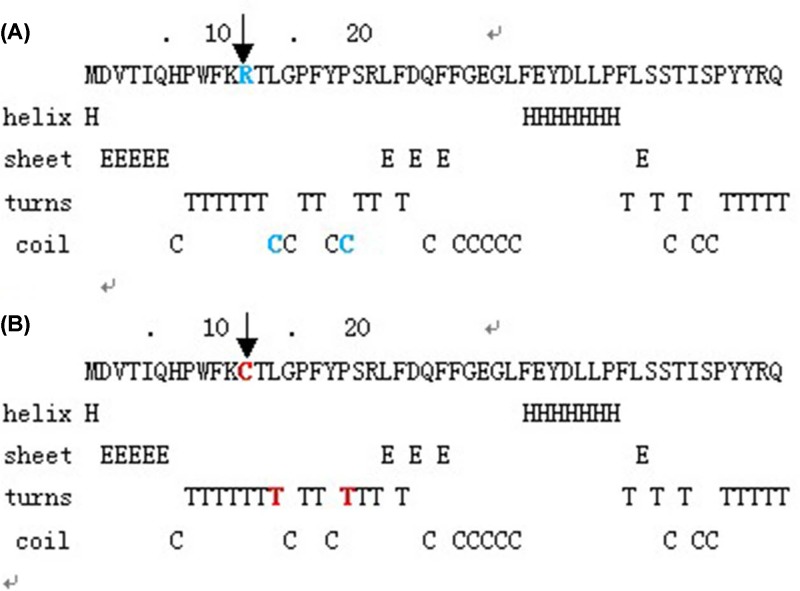
The effect of the mutation on the secondary structure of *CRYAA* (**A**) The structure of the wild-type. (**B**) The structure of mutant type. Using GOR method, the mutant CRYAA 12C replaced one coil “C” with “T” at amino acid 14 and a coil “C” with a turns “T” at position 19.

The place where the mutation occurred was located within a phylogenetically conserved region by multiple-sequence alignment (Figure 4). The comparison of hydrophobicity between wild-type and mutant type, and hydrophobicity of the mutant protein increased between 8 amino acids and 16 amino acids (Figure 5). Using the GOR method, the results for secondary structure prediction suggested that the mutant CRYAA 12C replaced one coil “C” with one turns “T” at amino acid 14 and a coil “C” with a turns “T” at position 19 (Figure 6).

## Discussion

In the present study, we identified a novel mutation (c. 34C>T) in *CRYAA* in a three-generation Chinese family with congenital cataract and myopia. This variation seemed to be the disease causative factor as it co-segregated with the disease phenotype in all affected individuals and was absent in unaffected family member and100 control subjects.

Crystallins are the major structural proteins in lens accounting for nearly 90% of total soluble proteins [11]. The crystallin superfamily is composed of α, β and γ-crystallins, which contribute to the maintenance of lens transparency and a proper refractive index of the lens [12]. *CRYAA* is a major structural protein of the lens, and is required for maintenance of lens transparency. It is also a member of the small heat-shock-protein (sHSP) family, which consists of stress-induced proteins, and has chaperone activity [13,14]. *CRYAA* contains a conserved α-crystallin domain, which is flanked on either side by a hydrophobic NH2-terminal domain or a hydrophilic unstructured COOH-terminal [15,16]. The COOH-terminal domain was important for substrate-binding ability and chaperoning activity [17]. Previous research has also shown that *CRYAA* may play a possible role in stimulating epithelial cell differentiation in the lens [18]. Furthermore, a knockout mouse strain with an αA crystallin gene deletion shows microphthalmia and eventual opacity of the lens, which demonstrates the important role of alpha A crystallin in the development and maintenance of lens transparency [19].

In our study, the mutation led to an amino acid substitution, R12C, close to the NH2-terminus of *CRYAA*. The multiple-sequence alignments showed that Arg12 was a highly conserved residue. The NH2-terminus was reported to assist protein oligomerization, stability, and substrate binding [20]. Changes in the NH2-terminus would greatly affect the function of the protein including reducing chaperoning activity. Furthermore, the bulky polar amino acid, arginine, plays an important role in maintaining the structural integrity of the *CRYAA* protein and oligomeric assembly [21]. Loss of arginine would cause a loss of positive charge and lead to abnormal folding. Functional studies have shown that loss of arginine destabilizes *CRYAA*, reduces its interactions with substrate proteins and chaperoning activity [22]. The phenotype of cataract is presumed to be caused by the reduced *CRYAA* molecular chaperoning ability on other lens proteins [23].

Up to now, a total of 11 mutations in *CRYAA* have been reported to cause inherited cataract (Table 3). Among them, five mutations are associated with cataract-microcornea syndrome (CMCC), *R12C* [9], *R21W* [8], *R54C* [24], *R116C* [25], and *R116H* [8,26], which are all located in the highly conserved arginine residues in the two major functional domains of the αA-crystallin, the NH2-terminus and the COOH-terminus. In 2007, Hansen et al. [8] first reported the mutation of R12C, which was associated with cataract-microcornea syndrome in a Danish family, showing posterior polar opacity progressing to dense nuclear and laminar cataract. In 2009, Zhang et al. [9] found the same mutation causing non-progressive CMCC in a Hong Kong family from China, which exhibits an altered heat-shock response. This mutation nuclear phenotype, with or without microcornea has also been reported in other three families [20,27,28]. Interestingly, all of them were clinically different from our study, which was associated with isolated lamellar cataract with myopia, but without microcornea. These studies have demonstrated that Arg at the 12th locus of the peptide is a mutation hotspot, which is the essential role causes congenital cataracts.

**Table 3:** Summary of identified mutation in *CRYAA*

Nucleotide	Amino acid	Phenotype	Inherited mode	Reference
c.346C>T	R116C	Congenital zonular central nuclear, some with microcornea	AD	[20]
c.27G>A	W9X	Congenital nuclear	AR	[21]
c.145C>T	R49C	Sporadic nuclear, with fundus	AD	[22]
c.62C>G	R21L	Hypoplasia presenile progressing from	AD	[23]
c.247G>A	G98R	Lamellar to total progressing, nuclear,	AD	[24]
c.34C>T	R12C	With or without microcornea	AD	[24]
c.130C>T	R21W	Central and laminar with Varying anterior and posterior Polar components	AD	[8]
c.347G>A	R116H	Nuclear with polar and/or equatorial ramification	AD	[8,25]
c.230C>T	R54C	Nuclear, with microcornea	AD	[26]
c.161G>C	R54P	Y-suture	AD	[27]

Myopia is the most common visual problem in the world. The main causes are environment and genetics [29]. A number of evidence reveal the importance of genetic factors in the development of myopia, although environmental factors such as near work and a city lifestyle appear to have a great impact on prevalence of myopia. Recent genome wide linkage studies have provided evidence of susceptibility loci for mild, moderate, and high myopia [29–31]. In this family, myopia is not random. The diagnosis is through strigent standards, and the affected members of this family live in the countryside where myopia is rare compared with urban populations [29,32–35]. Most myopia begins at school age except for hereditary high myopia [32].

The c.34C>T substitution observed in the present study caused the replacement of glycine to glutamic at codon 12 (p. R12C), localized in the first exon of *CRYAA*. The result of multiple-sequence alignments showed that Arg12 was a highly conserved residue. To investigate the effect of R12C substitution on *CRYAA* chaperoning activity, we use the GOR method, the secondary structure of the mutant type has been changed. Protein analysis by ProtScale clear showed that substitution in the *CRYAA* protein would increase the hydrophobicity between amino acids 8 and 16. These data indicate that the critical function of arginine. Mutation may have a detrimental physiological effect. As reported in other lens proteins, hydrophobicity is associated with crystallin activities, such as *CRYBB2* and *CRYGD* [36–38]. For *CRYAA*, hydrophobicity is involved in protein oligomerization and chaperoning activity [39,40]. Thus, the predicted increase of hydrophobicity around Arg-Cys substitution site might change the local protein structure and function.

There were also some limitations in the present study. First, the mutation has not been proved to be pathogenic in the present study. Further research using animal models to confirm the pathogenicity of the newly discovered disease sites was needed. Second, only one family data were analyzed in the present study. Further study with larger sample size was needed.

In conclusion, an autosomal dominant isolated lamellarl cataract associated with myopia in a Chinese family was described. Studies of functional protein, especially in a larger group of patients with mutations of *CRYAA* gene, will be very valuable in confirming typical reasons of *CRYAA*-related cataract. Research on *CRYAA* gene variations in populations with or without myopia would be very helpful in illustrating the role of *CRYAA* alterations in multifactorial myopia. Our finding confirms the high rate of apparently independent mutations at this dinucleotide.

## Data Availability

All data generated or analyzed during this study are included in this published article. The additional datasets used and/or analyzed during the current study are available from the corresponding author on reasonable request.
